# Chronic non-bacterial osteomyelitis: a comparative study between children and adults

**DOI:** 10.1186/s12969-019-0353-2

**Published:** 2019-07-23

**Authors:** Andrea Skrabl-Baumgartner, Peter Singer, Theresa Greimel, Gregor Gorkiewicz, Josef Hermann

**Affiliations:** 1 Division of General Pediatrics, Department of Pediatrics and Adolescent Medicine, Medical University Graz, Graz, Austria; 2 Diagnostic & Research Institute of Pathology, Medical University Graz, Graz, Austria; 3 Division of Rheumatology and Immunology, Department of Internal Medicine, Medical University Graz, Graz, Austria

**Keywords:** CRMO, SAPHO-syndrome, Chronic non-bacterial osteomyelitis, Spondyloarthritis

## Abstract

**Background:**

To compare clinical presentation, diagnostic and treatment strategies, and outcome between pediatric and adult patients with chronic non-bacterial osteomyelitis (CNO).

**Methods:**

Retrospective single-centre comparative study of pediatric and adult patients diagnosed with chronic recurrent multifocal osteomyelitis (CRMO)/CNO or synovitis, acne, pustulosis, hyperostosis, and osteitis (SAPHO) syndrome treated at the Medical University of Graz.

**Results:**

24 pediatric patients diagnosed with CRMO/CNO and 10 adult patients diagnosed with SAPHO syndrome were compared**.** Median age at diagnosis was 12.3 years (range 7.9–18.9) in the pediatric group and 32.5 years (range 22–56) in the adult group. Median time to diagnosis was shorter in children than in adults (0.3 vs. 1.0 years). Initial clinical presentation, laboratory and histopathological findings were similar in children and adults. Mean numbers of bone lesions were comparable between pediatric and adult patients (3.1 vs. 3.0), as were rates of skin involvement (33% vs. 30%). Sternal involvement was more frequent in adults whereas involvement of clavicle and long bones was more frequent in children (41.7% vs.10, 33% vs. 10%). Computerized tomography (CT) was used more often in adults, whereas whole-body magnetic resonance imaging (MRI) was used only in children. Bisphosphonates were applied more often in children and outcome was better in children than in adults (62.5% vs.30%).

**Conclusion:**

Results of our study suggest that CNO/CRMO and SAPHO syndrome in children and adults might represent a single clinical syndrome that needs a similar diagnostic and therapeutic approach.

## Background

Chronic non-bacterial osteomyelitis (CNO), an autoinflammatory disease characterized by sterile bone lesions, affects children and adults. In 1972 Giedion first described children with CNO and Björksten and colleagues in 1978 introduced the term chronic recurrent multifocal osteomyelitis (CRMO), to date the preferred term in pediatric patients [1, 2]. Clinical presentation varies widely, from mild, unifocal, and time-limited disease to severe, chronically active or recurrent disease with multifocal bone lesions accompanied by cutaneous manifestations and additional chronic immune-mediated diseases [2–5]. Consequently, terms such as chronic non-bacterial osteomyelitis (CNO) and nonbacterial osteitis (NBO) were introduced [6, 7]. In adults the term synovitis, acne, pustulosis, hyperostosis, and osteitis (SAPHO) syndrome is commonly used [8]. Whether CRMO/CNO in children and SAPHO syndrome in adults are a single disorder, and which term might best embrace both, remains unresolved [9].

Although CNO is currently recognized more frequently than in the past, misdiagnosis and delays in treatment persist for both children and adults, accompanied by exposure to radiation, biopsy procedures and prolonged antibiotic treatment. Reliable diagnostic criteria and treatment protocols are lacking. Studies comparing the diagnostic approach and management of CNO between pediatric and adult patients are scarce [5, 9]. To improve our understanding of the disease we sought to compare clinical presentation, biomarker values and radiological findings, as well as diagnostic work-up, treatment strategies, and outcome, between cohorts of pediatric and adult patients with CNO diagnosed and treated at one institution. We now share our findings and interpretations.

### Patients and methods

Patients diagnosed with CRMO/CNO or SAPHO syndrome between March 2004 and September 2016 with follow-up of at least 6 months were studied. All patients were diagnosed and treated at the Departments of Pediatrics and Adolescent Medicine, Rheumatology and Immunology, or Orthopedic surgery at our institution. Data, collected anonymously by reviewing electronic medical charts, included

1. Patient history, with age at onset of symptoms, initial symptoms, number of medical consultations before diagnosis, imaging assessments, biopsy procedures, and treatment before diagnosis.
2. Characteristics at diagnosis, with clinical presentation, including pain, swelling, local inflammatory signs, fever**,** arthritis and associated diseases; laboratory findings, including leukocyte count, C-reactive protein (CRP) and erythrocyte sedimentation rate (ESR) values, antinuclear antibody (ANA) titers, and HLA-B27 status; radiologic findings, including number and distribution of inflammatory bone lesions; and histopathologic findings on bone biopsy.
3. Treatment and response, with remission defined as clinical absence of manifestations, normalization of inflammatory-marker values, and radiologic absence of active bone lesions.
4. Course of disease, with “relapsing course” defined as repeated flares interspersed with symptom-free periods during which no treatment was given and “persistent course” as complaints continuing for at least 6 months, with or without treatment
5. Outcome and complications.

### To compare histopathologic results, bone specimens were reevaluated by a histopathologist (G.G.)

#### Patients in whom symptoms had started before age 18 years were assigned to the pediatric group

Statistical analysis: Continuous variables were depicted in mean ± SD or median (IQR or range) depending on normal distribution. Distribution of leukocyte counts, CRP, and ESR-levels in children and adults were depicted as boxplots. All statistical analyses were performed using GraphPad Prism 6.0 (Graphpad, San Diego, CA).

The study was approved by the local ethics committee (EK 29–337 ex 16/17). The institutional review board did not request patient’s written informed consent.

## Results

### Patient characteristics

We studied 34 patients (24 pediatric and 10 adult) with a median follow-up of 4.8 years in pediatric and 4.3 years in adult patients (Table 1).

**Table 1:** Characteristics of pediatric (*n* = 24) and adult (*n* = 10) patients with CNO

Variable	Pediatric patients, Value	Adult patients, Value
Demographic characteristics		
Female, *n* (%)	14 (58.5%)	6 (60%)
Age at diagnosis, yrs., median (range)	12.3 (7.9–18.9)	32.5 (22–56)
Delay in diagnosis, yrs., median (range)	0.3 (0–12.9)	1.0 (0.4–17.1)
Clinical characteristics		
Arthritis	12 (50%)	3 (30%)
- Arthritis distant to bone lesion	4 (16.7%)	1 (10%)
- Sacroiliacal involvement, bilateral	6 (25%), 4 (16.7%)	1 (10%), 0
Associated diseases	10 (41.5%)	5 (50%)
- skin involvement	8 (33.3%)	4 (40%)
- severe acne	7 (29.2%)	1 (10%)
- pustolosis palmoplantaris	0	2 (20%)
- psoriasis vulgaris	1 (4.2%)	0
- hidradenitis suppurativa	0	1 (10%)
- ocular involvement	3 (12.5%)	1 (10%)
- coeliac disease	1 (4.2%)	0
Radiological characteristics		
Number of radiological lesions, mean	3.1	3.0
median (range)	2 (1–7)	2 (1–9)
- unifokal	10 (41.7%)	4 (40%)
Clavicular involvement, sole	10 (41.7%), 7 (28.3%)	1 (10%), 0
Sternal involvement	2 (8.3%)	5 (50%)
Long bone metaphysis involvement	8 (33.3%)	1 (10%)
Vertebral involvement	5 (20.8%)	5 (50%)
Histological characteristics		
Fibrosis, *n* (%)	16/16 (100%)	6/6 (100%)
Chronic inflammation, *n* (%) (Lymphocytes, plasmocytes, histiocytes)	16/16 (100%)	6/6 (100%)
Acute inflammation, *n* (%) (Neutrophilic granulocytes)	8/16 (50%)	2/6 (33.3%)

Median age at onset of symptoms was 11.8 years (range 6.3–17.9) in the pediatric group and 28.3 years (18.1–54.2) in the adult group. Median age at diagnosis was 12.3 years (range 7.9–18.9) in the pediatric group and 32.5 years (22–56) in the adult. Median time to diagnosis was shorter in the pediatric group than in the adult (0.3 vs. 1.0 years respectively). In both groups females (58.5% vs. 60%) predominated. The number of physicians consulted before diagnosis was 2.1 (range 1–3) in children and 2.5 [1–4] in adults. 37.5% of pediatric patients had a family history of chronic immune-mediated diseases in first- or second-degree relatives, including CNO (in a monozygotic co-twin sister), compared with 20% of adult patients.

### Clinical presentation

95.9% of the pediatric and 100% of the adult patients reported pain as a first symptom, accompanied by local swelling in 79.2% of children and 50.0% of adults. Local signs of inflammation (heat, redness) were reported in 50% of children and 10% of adults. None of the patients presented with fever.

*Associated diseases* were found in 41.5% of pediatric and 50% of adult patients, with skin lesions predominating in both groups (8 children and 4 adults, 33.3% vs. 40% respectively). Severe acne was most frequent in children (7, 29.2%), whereas palmoplantar pustulosis (PPP) was most frequent in adults (2, 20%), (Table 1). Skin disease preceded the diagnosis of CNO in 16% of pediatric patients and 30% of adult patients and appeared after diagnosis of CNO in respectively 16 and 10% of pediatric and adult patients within 2 and 7 years.

Ocular involvement was found in 12.5% of children and 10% of adult patients. One child had coeliac disease.

### Imaging assessments

X-ray images were performed in all patients and demonstrated abnormalities in 69.6% of pediatric patients and 75% of adult patients. MRI of clinically overt lesions was conducted in all pediatric and adult patients. CT imaging was conducted more frequently in adult patients than in pediatric patients (80% vs.12.5%). Imaging techniques used to detect additional lesions differed between children and adults. Whereas whole-body MRI was performed solely in children (21%), technetium bone scans were deployed more frequently in adults (80% vs. 58.5%). Osteolytic lesions were seen in 30.4% of pediatric and 50% of adult patients and hyperostotic lesions in 30.4% of pediatric and 10% of adult patients.

**Fig. 1:**
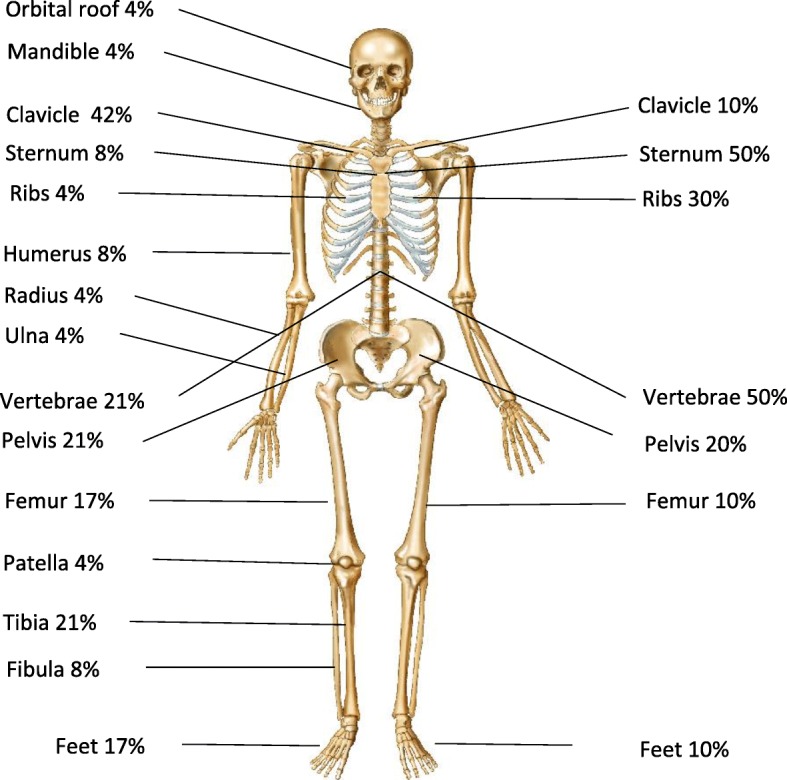
Distribution of bone lesions in patients with CNO; left side showing pediatric patients, right side showing adult patients

Mean numbers of bone lesions were comparable between the groups, with 3.1 in children (range 1–7) and 3.0 in adults [1–9]. Unifocal involvement did not differ between pediatric and adult patients (41.7% vs. 40%). Sites of lesions differed, however, with involvement of clavicle (pediatric 41.7% vs. adult 10%) and long-bone metaphyses (pediatric 33.3% vs. adult 10%) more frequent in children. In contrast, involvement of the sternum was more frequent in adults (pediatric 8.3% vs. adult 50%), as was involvement of the vertebrae (pediatric 21% vs. adult 50%). Figure 1 presents the distribution of bone lesions.

*Associated arthritis* was found in 50% of pediatric and 30% of adult patients; the joints involved mainly adjoined bone lesions (Table 1).

*Overlapping features with SpA* were observed in 6 pediatric and one adult patient (25% vs. 10% respectively) of whom 5 were males. All patients demonstrated involvement of the sacroiliac joint. Sacroiliitis was present at time of diagnosis of CNO in all 7 patients. Only one of 6 tested patients was HLA-B27 positive. Table 2 shows the characteristics of this subgroup of CNO patients.

**Table 2:** Characteristics of CNO patients with overlapping features of spondyloarthritis

Pat	Gender	Age at onset (yrs)	HLA-B27	Sacroileitis	Peripheral arthritis	Skin affection	Uveitis anterior	FH	Response to TNFα-antagonist
6	m	12.22	pos	bilateral	yes	SA	yes	no	yes
11	m	16.7	neg	unilateral	no	SA	no	no	yes
27	m	6.3	neg	bilateral	no	SA	yes	no	–
28	w	7	n.d.	unilateral	yes	no	no	yes	yes
30	w	18.2	neg	unilateral	yes	PPP	no	no	–
31	m	17.8	neg	bilateral	yes	SA	no	no	–
33	m	14.3	neg	unilateral	yes	SA	no	no	yes

*FH* family history, *PPP* palmoplantar pustulosis, *SA* severe acne, *n.d.* not done

*Bone biopsies* were performed at least once in 19 children (79.4%) and 9 adults (90%) to exclude infectious osteomyelitis and malignancy. Histopathologic results at initial presentation did not differ between the two groups concerning inflammation and fibrosis of the bonemarrow (Table 1).

Results of bacterial culture were available in 17 children and 7 adults. Amplification of 16S ribosomal (r) RNA was performed in 6 children and 2 adults. Mycobacterial PCR was performed in 2 children and one adult. Bacterial pathogens were detected in 3 patients: One biopsy specimen grew *Staphylococcus epidermidis* and *Propionibacterium acnes* on enrichment-culture taken from sternal lesion in an adult patient with severe acne. PCR revealed *Staphylococcus epidermidis*, *Neisseria* sp., and *Streptococcus oralis* in one child’s tibial lesion, and *Enterobacter klebsiella* at low bacterial load in the second rib of an adult. Bacterial culture yielded no organisms in either the tibial or the second-rib lesion. All detected organisms were interpreted as contaminants.

**Fig. 2:**
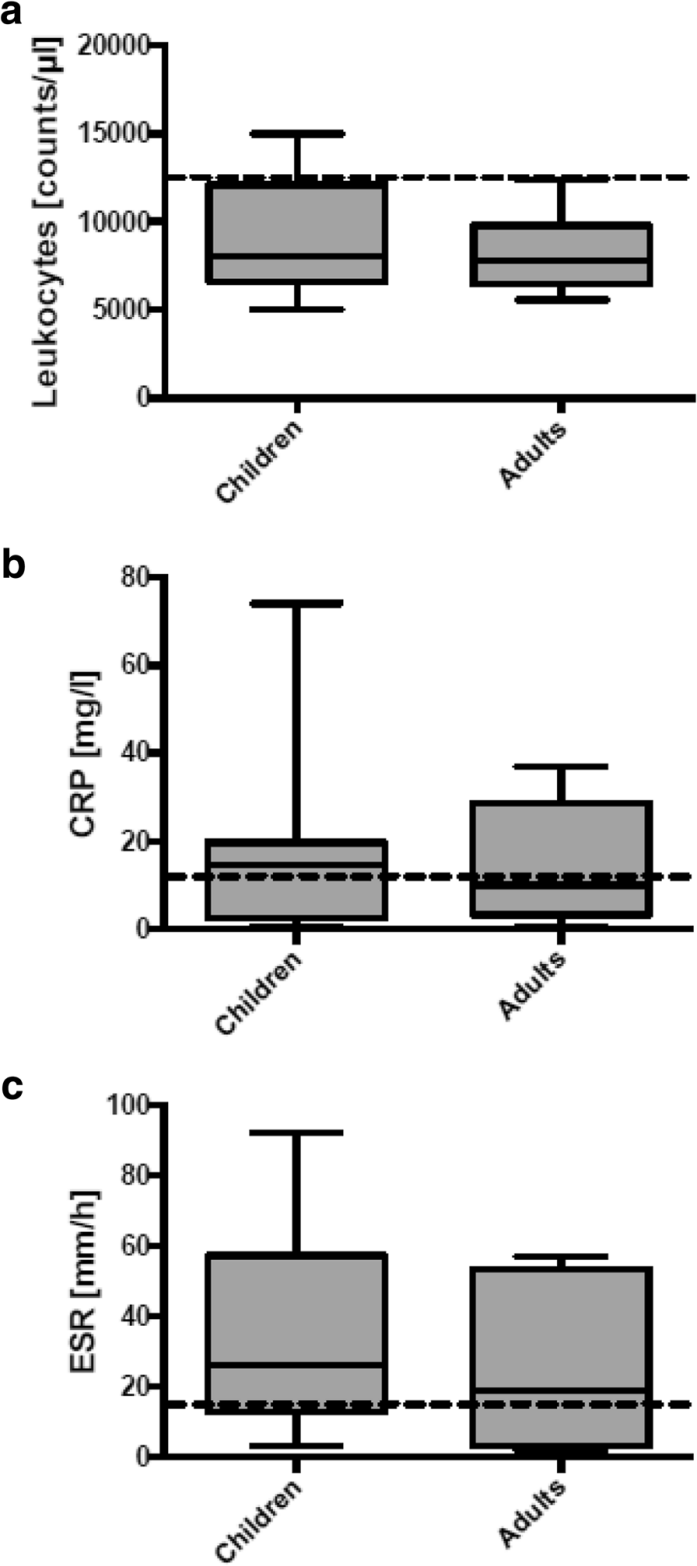
Laboratory data of children and adults with CNO: **a** leukocytes, **b** C-reactive protein (CRP), **c** erythrocyte sedimentation rate (ESR). Broken lines depict upper normal values

*Laboratory tests:* 20.8% of pediatric but no adult patient exhibited leukocytosis. 66.7% of pediatric and 50% of adult patients presented with elevated C-reactive protein values > 5 mg/l. 63.7% of pediatric and 57% of adult patients had ESR values > 15 mm/hour (Fig. 2). The HLA-B27 phenotype was found in 3 of 15 tested children (20%) and in none of 5 tested adults; it occurs in 8% of inhabitants of our geographical region. [10]. ANA were demonstrated in 3 of 21 tested children (14.3%), with titers ranging between 1:320–1:5120, but were absent in 3 adults tested.

### Treatment before diagnosis

Starting with a working diagnosis of bacterial infection, 62.5% of pediatric and 55.6% of adult patients received intravenous and/or oral antibiotics with a mean duration of 4 weeks (range 0–25) in children and 5.1 weeks (range 0–26) in adults. Further treatment strategies in children included immobilization with plaster casts in 5, prostaglandin-E infusions in 3, hyperbaric oxygenation in 2, and surgical resection in one. Four adults underwent therapy with prostaglandin-E, gabapentin, surgical resection, or high-frequency radio ablation (one patient each).

### Treatment after diagnosis

Non-steroidal anti-inflammatory drugs (NSAIDs) were the most common first-line treatment, used in 83.3% of pediatric patients and all adults. 75% of children, but only 20% of adults, received NSAIDs continuously. Remission was observed in 3 of 20 children (15%) and in one adult (10%). Systemic steroids were used as a second-line treatment in 29.2% of children and 20% of adults, with transient response in all but one child. Topical steroids were used in one patient from each group (4.2% vs. 10%). Further treatment included disease-modifying anti-rheumatic drugs (DMARDS), which were used in 2 (8.3%) children and 3 (30%) adults. Children were treated with either methotrexate (MTX) or sulfasalazine (SSZ). One adult patient was treated with MTX, a second was treated with SSZ first-line, followed by MTX, and a third was treated with MTX first-line followed by leflunomide and than by cyclosporine A. Remission was observed in only one adult patient, who received MTX. The use of bisphosphonates differed between groups. These drugs were administered in 13 children (54.1%) but only one adult (10%). Bisphosphonate treatment led to remission in 9 of 13 children (69.4%) and in the adult patient. Another difference was seen in biological treatment. Children received tumor necrosis factor (TNF)-α-antagonists twice as often as adults (20.8% vs. 10%) with remissions seen in 3 of 5 children (59.9%), but not in the adult. Table 3.

**Table 3:** Patient treatments and outcomes, children (*n* = 24) and adult patients

Variable	Pediatric patients	Adult patients
Treatment		
Antibiotic treatment, *n* (%)	15 (62.5%)	5/9 (56%)
- Duration, weeks, mean (range)	4 (0–25)	5.1 (0–26)
NSAID, *n* (%)	20 (83.3%)	10 (100%)
- NSAID continuously, n (%)	18 (75%)	2 (20%)
Steroids, *n* (%)	8 (33.3%)	3 (30%)
- systemic, *n* (%)	7 (29.2%)	2 (20%)
DMARDS, *n* (%)	2 (8.3%)	3 (30%)
- Sulfasalazine, *n*	1	1
- Methotrexate, *n*	1	3
- Leflunomide, *n*	0	1
- Cyclosporine A, *n*	0	1
Anti-TNF, *n* (%)	5 (20.8%)	1 (10%)
Bisphosphonates, *n* (%)	13 (54.1%)	1 (10%)
Final outcome		
Remission, *n* (%)	15 (62.5%)	3 (30%)
Complications, *n* (%)	8 (33.3%)	6 (60%)
Fracture	2 (8.3%)	3 (30%)

### Outcome and complications

The course of disease was chronic and remittent in the majority of children (57%), whereas in adults it was predominantly chronic (83%).

At last follow-up visit 62.5% of pediatric patients and 30% of adult patients were in remission. Complications were observed in 33.3% of pediatric patients and 60% of adults. Complications in children included fracture of the vertebra in 2, bone deformity in 1, and chronic pain in 2. Complications in adults were fracture of a vertebra and chronic pain (3 patients each, no overlap). Fracture rates were 8.3% in children and 30% in adults. (Table 3).

## Discussion

By comparing a pediatric and adult population of patients diagnosed with CRMO/CNO or SAPHO syndrome, our study showed that these two diseases share most clinical features but differ in few noteworthy aspects.

Although we found no difference between the groups in respect of unifocal and multifocal involvement, we found age-related predilections for certain anatomic sites. In accordance with a study by Greenwood et al. [9], upper anterior chest wall and spinal involvement were the most frequent affected sites in adult patients. By contrast, we found involvement of the clavicle the most common affected site in children, followed by long bone metaphyses. Interestingly, unifocal clavicular involvement was seen only in children, in line with a description of the swollen clavicle as a classical lesion in CRMO in children.

Another clinical difference was found in regard to skin involvement. Skin affections were diagnosed more often in adults, with a similar frequency as seen by Girschick et al., who found dermatologic manifestations in 41% of 31 adult patients and in 19% of 455 pediatric patients [5]. In our cohorts, PPP was the most frequent manifestation in adults and severe acne was the most frequent in children. In accordance with our findings, PPP was reported as the leading skin disorder in the adult cohort of the Eurofever registry and Schnabel et al. reported that acne fulminans was the principal disorder in their pediatric cohort [11]. Skin disease developed after presentation with osteoarticular lesions in 42% of our patients, making a diagnosis of CNO more difficult, especially in the presence of unifocal bone disease.

We observed SpA-like features in 7 of our patients. The 6 pediatric patients fulfilled the International League of Associations for Rheumatology criteria for the enthesitis-associated arthritis subtype of JIA. The adult patient fullilled the criteria for the Assessment of Spondyloarthritis International Society classification criteria for SpA [12]. This observation is in line with results published by Kaiser et al., who described features of SpA in 21% of patients of their cohort concluding that a spondylarthritis subgroup of CNO exists [13]. Vittecoq et al. have also discussed the overlap between CNO and SpA, suggesting that CRMO is an unusual form of SpA [14].

Sacroiliacal involvement, usually unilateral, is well recognized in CNO, affecting 13–52% of cases in children as well as in adults. [13–16]. Sacroiliitis was found in all of our SpA-like 7 patients, with unilateral involvement in 4 and bilateral in 3 patients. Only one of 6 tested patients had the HLA-B27 haplotype, in line with results of other studies [5, 13, 14]. Sacroiliitis was present at diagnosis of osteitis in all of our patients, in line with data reported by Kaiser et al., but differing from those of Vittequoc et al, who described evolution of CNO towards SpA over time in all but one of their patients [14].

Additional variations seen in the cohorts investigated differed only by the diagnostic and therapeutic approaches of the treating physicians.

CT as well as technetium radioimaging were deployed less frequently in children, to avoid radiation, which has been demonstrated a risk for malignancies especially in these [23]. Alternative procedures, such as MRI have become increasingly important. Interestingly, in our study population whole-body MRIs were performed only in children. MRI was demonstrably superior to technetium bone scans in the detection of silent lesions on initial assessment as well as in monitoring response in children [17, 18]. In adults, whole-body scintigraphy is commonly used to detect silent bone lesions [9, 19] probably because studies investigating the value of whole-body MRI in SAPHO syndrome are lacking and, insurance companies in our country do usually not cover whole-body MRI .

Treatment with bisphosphonates varied substantially between adults and children. Since bisphosphonates have demonstrated efficacy in reducing pain, inhibiting inflammation, and even in remodelling of vertebrae in several studies in children [20–22], bisphosphonates are often used early in those with vertebral lesions. Although vertebral involvement in our cohort was more common in adults, only one of these received bisphosphonates. By contrast, more than half of the pediatric patients received bisphosphonates as second-line treatment early in disease, which may have contributed to better outcome and lower rates of complications including bone fractures. Only one investigation looked at the effect of bisphosphonates in adult patients with SAPHO syndrome. Eight of 14 patients demonstrated response, causing the authors to describe bisphosphonates as a noteworthy treatment option in adults of which treating physicians are not aware of [24].

Four patients of our spondyloarthritis sub-cohort received anti-TNF therapy. All responded favorably to etanercept, which was given after failure of NSAIDS and conventional DMARD in all and, in addition, to bisphosphonates in one patient. Further studies are needed to identify CNO patients who would benefit from anti-TNF rather than from bisphosphonate therapy as second-line treatment.

## Conclusion

Clinical appearance and imaging-study, histopathologic, and biomarker findings are similar in pediatric and adult patients diagnosed with CRMO/CNO or SAPHO syndrome in our study indicating, that CRMO/CNO and SAPHO syndrome might be variant phenotypes of the same entity. Discovery of the spondylarthropathic subtype in CNO patients could prompt anti-TNF therapy. Diagnostic and treatment strategies in pediatric and adult patients vary considerably, pointing to the need for validated guidelines.

## Data Availability

All data generated or analyzed during this study are included in this published article.

## Abbreviations

CNO: Chronic non-bacterial osteomyelitis
CRMO: Chronic recurrent multifocal osteomyelitis; synovitis
SAPHO: Acne, pustulosis, hyperostosis, and osteitis syndrome
SpA: Spondyloarthritis
